# VLSI implementation of a modified min-max median filter using an area and power competent tritonic sorter for image denoising

**DOI:** 10.1038/s41598-024-80053-6

**Published:** 2024-11-19

**Authors:** Chrishia Christudhas, Annis Fathima

**Affiliations:** School of Electronics Engineering, Vellore Institute of Technology, Chennai, Tamil Nadu India

**Keywords:** Min-Max median filter, Tritonic Sorter, Image denoising, Impulse noise, ZedBoard Zynq, Vivado tool, Energy science and technology, Engineering

## Abstract

The prominence of image processing in today’s cutting-edge technology is undeniable. Integrating software with hardware leverages both strengths, resulting in a real-time processing system that is efficient and streamlined. Raw images are usually affected by noise, which hinders the acquisition of good-quality and detailed images; hence, denoising becomes necessary. This paper proposes a modified min-max median (MMM) filter to remove impulse noise and a Tritonic sorter to localize corrupted pixels. The proposed denoising method focuses on localizing noisy pixels, unlike traditional denoising approaches, which focus only on noise detection and filtering. A min-max sheet provides the location of the corrupted pixels, and filtering is performed on them. The Tritonic Sorter, consisting of a max locator and a min locator, compares three input values and finds the minimum, maximum and median values among them. Compared to other state-of-the-art methods, the proposed method minimizes the number of comparators needed to carry out the sorting process. The proposed method was synthesized in the ZedBoard Zynq kit using the Vivado tool. The results show that the area improved by 27%, and the power improved by 16.23% compared with those of the existing method.

## Introduction

The use of portable photographic devices such as cameras, smartphones, and tablets has resulted in an increasingly important shift in people’s routines. Currently, images play a fundamental role in human communication, education, and technology. Images are often subjected to noise. Noise can result from transmission channels, poor illumination, sensors, etc. Filters can eliminate noise while preserving fine details and information in the image with less distortion. In the literature, there are many filters available for noise reduction in images. The image filtering techniques are divided into spatial and frequency domains. The spatial domain is further subdivided into linear and nonlinear filters. The linear filters tend to blur the edges and are not successful in removing noise in the images; on the other hand, nonlinear filters preserve the edges and remove noise efficiently, more specifically, impulse noise. Impulse or salt and pepper noise is where the pixel cannot take the intended initial value but takes either of the extreme values, i.e., 0 or 255. The min filter, max filter, median filter, and a combination of both min-max median filters^1^ are nonlinear filters. The median filter has been modified, and various new versions of the filters are now available. Switching median filters^2^, adaptive switching median filters^3^, weighted median filters^4^, approximated median filters^5^, and hybrid median filters are some of the well-known modified versions of median filters. Although nonlinear filters have many advantages over linear filters, they are mainly application-oriented.

In today’s electronic market, software must incorporate hardware. The successful integration of software design into hardware garners is of interest to peers in the research community. It also gains interest from potential investors and buyers who recognize the potential of the product or device that could positively impact people’s lives. Hardware architecture design is tricky and more constrained than software design, as the design must be tested with a minimum failure rate. Field programmable gate arrays (FPGAs) are popularly used as accelerator platforms in various fields of image-processing applications^6,7] , [8^ where high performance, high energy efficiency, and low power are needed. Noise reduction filters usually require the rigorous use of arithmetic operations and comparisons, thus increasing the system’s resource usage. Therefore, low-power, area-efficient and high-performance approaches are necessary for very large-scale integration (VLSI) design to implement the most in-demand applications. The paper discusses the noise reduction technique used for images and its implementation in Vivado.

## Related works

The median filter is a common nonlinear noise reduction technique in image processing. Its importance today remains significant due to its effectiveness in removing impulse noise, preserving edges, and improving computational efficiency. It continues to be an essential tool for improving the quality of images in diverse applications across different domains. Various denoising techniques based on the median filter and its advanced techniques are discussed in^9^. Draz et al.^10^ analyze median filtering units connected to a general purpose processor (GPP) for FPGA-based systems applications. The algorithms proposed in the paper are tested for grayscale and color images and show reduced computational complexity. It is implemented in the Xilinx Virtex-5 LX110T board. The VLSI focuses mainly on speed, area, and power efficiency. Monajati et al.^11^ designed three imprecise two-bit magnitude comparators. A histogram-based error distortion plot is used to evaluate the error. The design was synthesized using the Synopsys Design Compiler with Nand gate 45 nm Open Cell Library. The simulation results showed that the quality of the output images is slightly less accurate.

Usually, a threshold is set prior to the conventional switching median filter; based upon that, median filtering^12^ is carried out. The threshold is computed locally from pixel intensity values in the window This technique preserves more details and performs better in terms of the peak signal-to-noise ratio (PSNR) and mean absolute error (MAE). An adaptive sequentially weighted median filter was proposed in^13^. The 3σ principle of normal distribution and local intensity statistics is used here. This technique achieved good results in terms of noise removal and edge preservation. Kim et al.^14^ proposed a method to remove a mixture of impulse and Gaussian noise. A one-dimensional image filtering technique is proposed in^15^ reduces memory utilization. This paper targets the removal of Gaussian noise. The proposed design achieves a delay of 3.396 ns and a power of 158mw. The filter’s accuracy is tested using PSNR and SSIM. It is implemented using ZYNQ-7000(XC7Z020CLG-484).

Various digital components, such as adders, multipliers and comparators, are used for nonlinear filtering techniques. Hence, efficient designs of the digital components for performing median filtering are discussed further. A fast-sorting technique for N-sorters and N-filters was proposed in^16^. The authors were able to improve the speed compared to other state-of-the-art methods. Their technique was implemented in an FPGA for both 7 series and Ultrascale devices. Kent et al. proposed a merge sorting technique^17^ that incorporated pipeline and parallel processing, thereby enhancing the speed. This technique was implemented in the Xilinx Ultrascale Series. An architecture to reduce noise with fewer comparators was implemented in^18^. This technique was targeted for device XCV10 0-bg560 on XILINX 7. The performance of median filtering without comparators is discussed in ^19^ and ^20^. A median filtering system using merged insertion sorting is discussed in^19^. This technique was implemented in Altera’s Cyclone series EP4CE6F17C8. Lu et al.^20^ implemented a sorting optimization algorithm that reduced the number of comparisons needed to perform median filtering for a 3 × 3 kernel size. The design was synthesized in Altera’s Cyclone II FPGA chip.

Muneer et al.^21^ designed a tone-mapping operator for real-time embedded applications. This design was implemented in graphics processing units (GPUs) and FPGAs; the FPGA yielded better power consumption and speed results. A face detection engine^22^ was designed and implemented in the FPGA. This design detects faces with high reliability in real-time. The low-power median filter proposed in^23^ reduces the area cost without compromising the speed. The proposed method was synthesized using 90 nm CMOS process technology. An inaccurate median filter (IAMF) proposed in^24^ proved area-efficient and yielded good-quality images. It was synthesized in the General Process Design Kit (GPDK) 90 nm library with a clock frequency of 500 MHz. Goel et al.^25^ designed and implemented a 2D median filter to obtain high throughput. A hardware implementation to detect blood groups using digital signal processing (DSP) techniques is discussed in^26^. This method was implemented in the FPGA to minimize the power factor and increase the speed. Elloumi et al.^27^ discusses a system implemented in an FPGA for image denoising. This system reduced the hardware cost and increased the speed. The architecture that integrates pipelining and parallel processing to perform speed-efficient median filtering was discussed in^28^. A core idea for designing a fast median filter was implemented in^29^ by reducing the number of comparators.

A literature survey revealed various median filter implementations using VLSI cores and paved the way for our research. This paper proposes a modified MMM filter to remove impulse noise, which finds the minimum, maximum and median values using a proposed Tritonic Sorter. This paper is arranged as follows: Sect. 3 proposes the MMM filter. The MMM filter using a proposed Tritonic Sorter is discussed in Sect. 4, followed by the experimentation analysis in Sect. 5 and the conclusion in Sect. 6.

## Proposed MMM filter

Equation (1) gives the general equation of the median filter and obtains the neighboring pixel values needed for filtering.

1 $$A_{med}(m,n)=med(a_{3 \times 3}[m-1:n-1:m+1:n+1])$$

On the other hand, the traditional min and max filters replace the target pixel value with the maximum or the minimum value in the window. The equation for the min/max filter is given by Eq. (2).

2 $$A_{3 {min}/{max}}(m,n)=min(a_{3 \times 3}[m-1:m+1:n-1:n+1])\parallel max(a_{3 \times 3}[m-1:m+1:n-1:n+1])$$

**Fig. 1:**
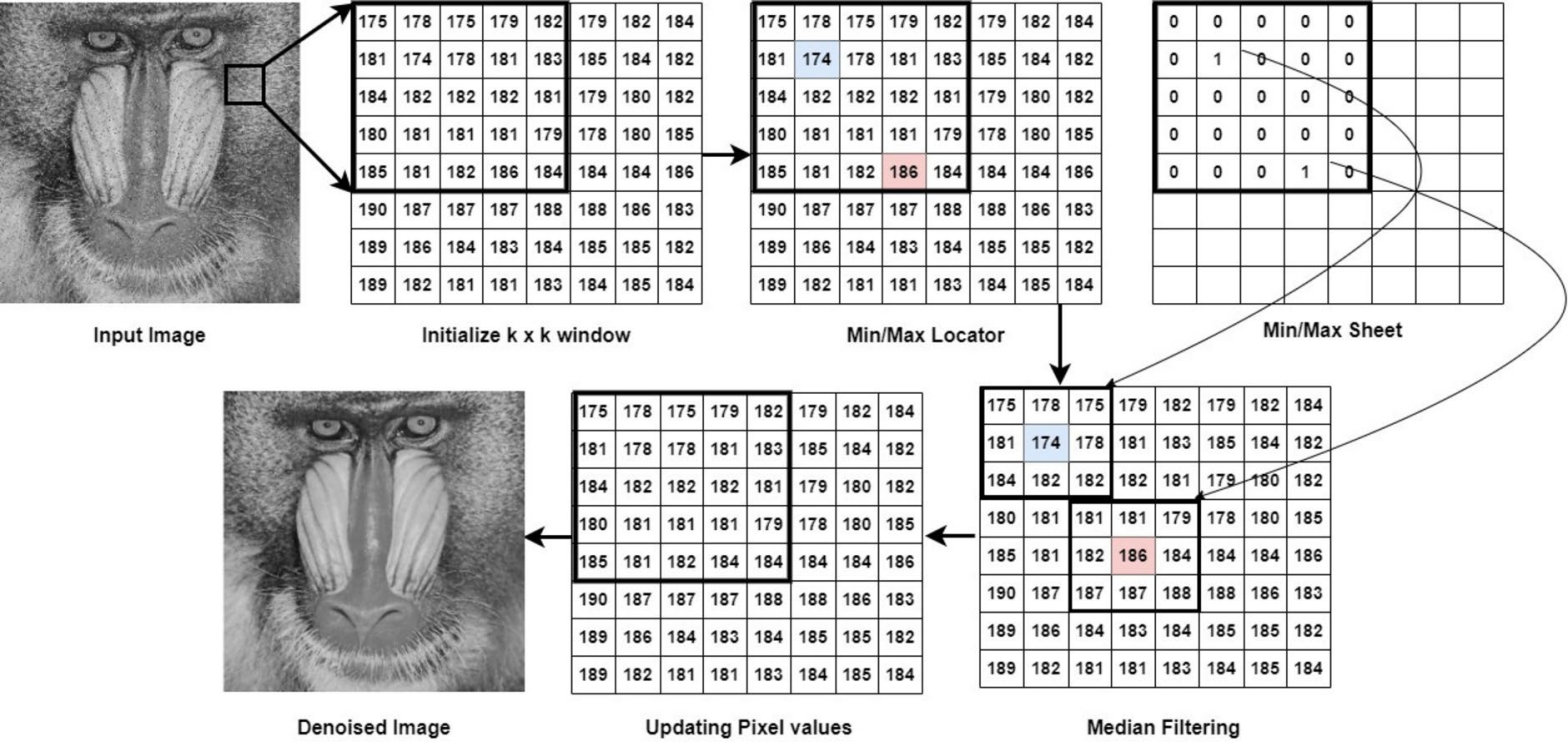
Flow diagram of the min-max median filtering.

The flow diagram of the proposed MMM filter is shown in Fig. 1. A k x k sliding window slides over the noisy image. The maximum and minimum pixel values in each window are identified and given a value of 1 in a separate min-max sheet. The purpose of the min-max sheet is to indicate where the min and the max values are located. This min-max sheet contains only binary values, where 1 indicates noisy pixels, and 0 indicates noise-free pixels. Once the min-max sheet is updated, all the locations where 1 is present are considered the target pixel, and that location is mapped to the original image. The neighboring pixel values are obtained, and median filtering is performed. Then, the median value is substituted for the target pixel value, and the denoised image is obtained.

**Fig. 2:**
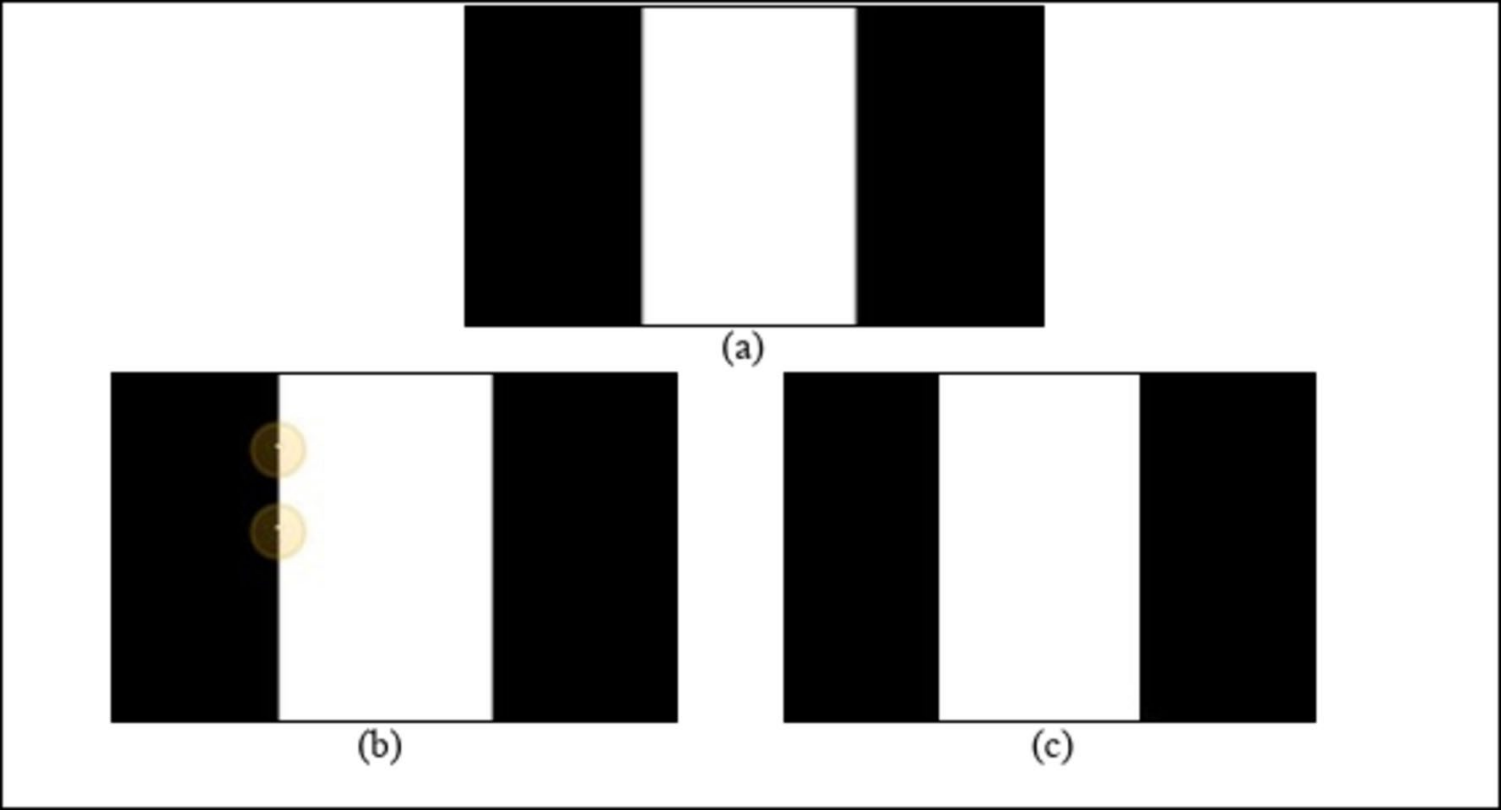
(**a**) Original image, (**b**) median filtered image (**c**) MMM filtered image

While the minimum, maximum and median filters preserve the edges, the minimum and maximum filters can cause a blurring, and the median filter can sometimes oversmooth the image. Figure 2 shows the edge preservation achieved by applying the median and MMM filters. The edges are better preserved, and the effect of blurring is negligible in the MMM filter approach compared with that in the median filter approach.

The equations for the proposed method are given by Eqs. (3) and (4). Equation (3) obtains the min/max values in each window. Equation (4) is the equation of the MMM filter.

3 $$\:{A}_{k\:min/max}\left(m,n\right)=\text{min}\begin{array}{c}\left({a}_{kxk}\left[m-\frac{k-1}{2}:m+\frac{k-1}{2},\:n-\frac{k-1}{2}:n+\frac{k-1}{2}\right]\right)\:\left|\right|\:\\\:max\:\left({a}_{kxk}\left[m-\frac{k-1}{2}\::m+\frac{k-1}{2},n-\frac{k-1}{2}\::n+\frac{k-1}{2}\right]\right)\end{array}\:$$

4 $$\:{A}_{MMM}(m,n)=\:{A}_{k\:min/max}\:\&\&\:{A}_{med}$$

## MMM filter with the proposed tritonic sorter

**Fig. 3:**
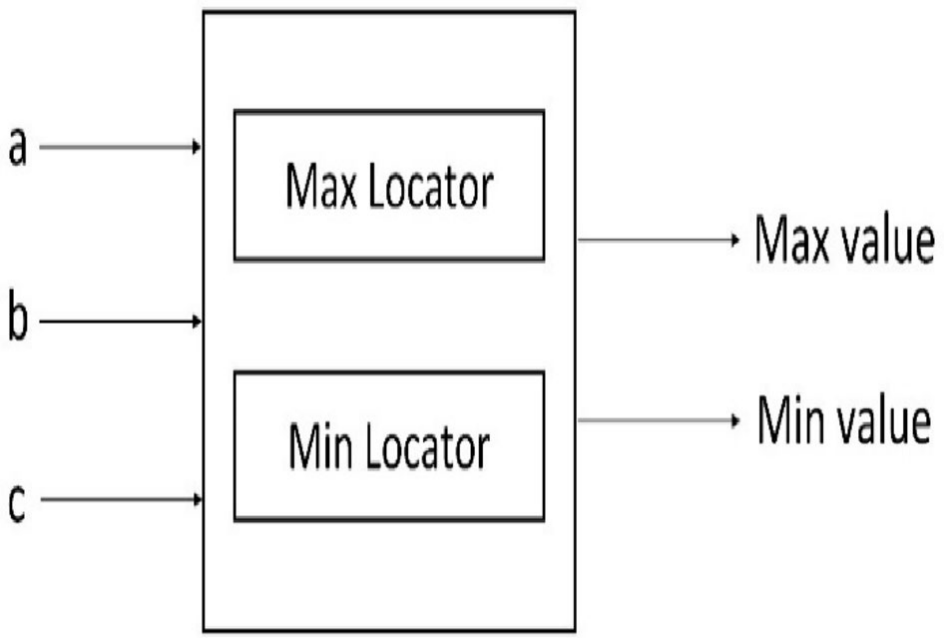
Tritonic sorter.

Implementing the proposed MMM filter in hardware requires efficient utilization of the resources. An efficient sorter is needed to obtain the minimum and maximum values in each window and to perform median filtering. The sorters widely used for sorting are parallel sorters, parallel merge sorters, odd-even sorters, and bitonic sorters. A parallel sorter needs 25 comparators to sort eight values from which the minimum, maximum and median values are obtained. The parallel merge sorter requires 19 comparators, the odd-even sorter requires 19 comparators, and the Bitonic sorter requires 24 comparators. A greater number of comparators corresponds to more memory being utilized. Hence, a novel Tritonic Sorter is proposed to reduce the number of comparators. The Tritonic Sorter is an extended version of the Bitonic Sorter. The Tritonic Sorter block, shown in Fig. 3, consists of a max and a min locator.

**Fig. 4:**
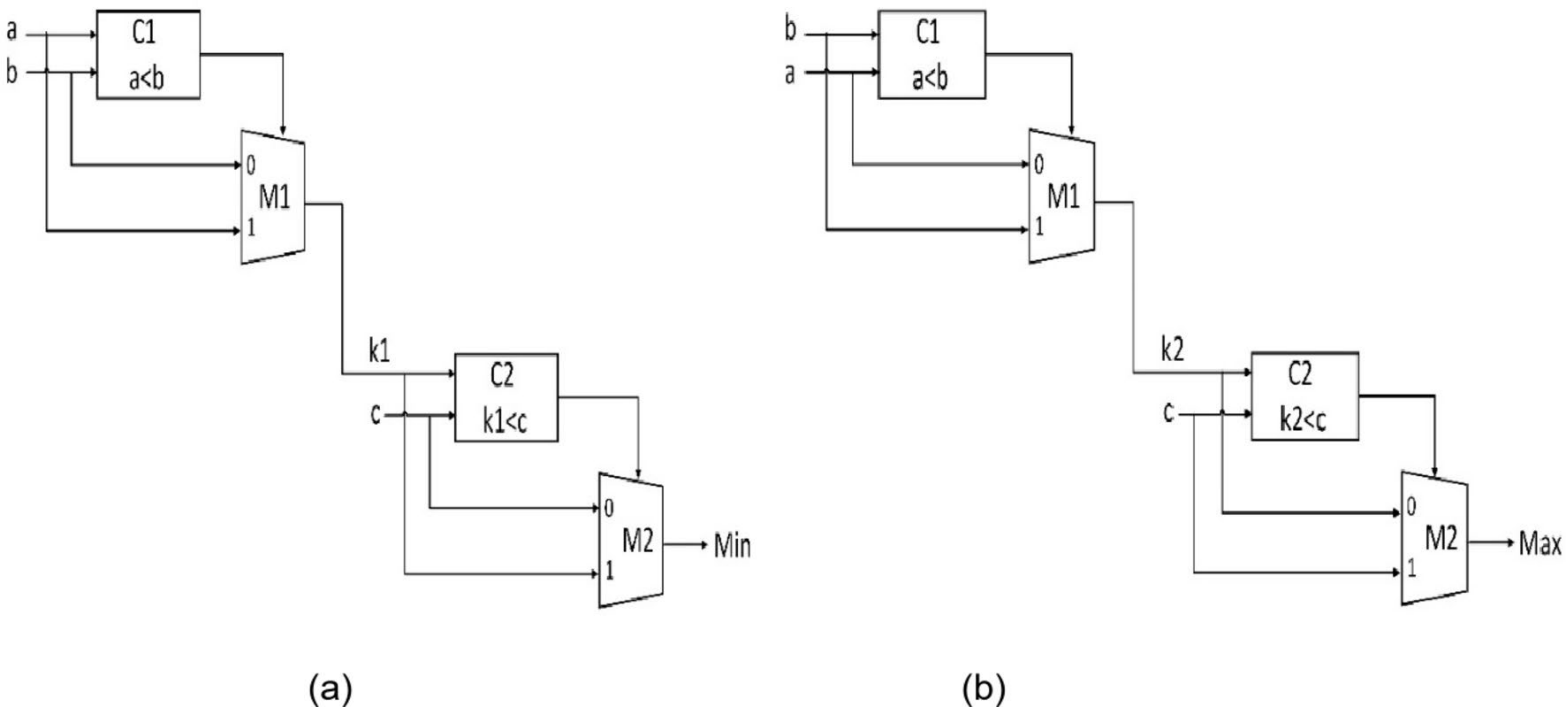
(**a**) Min Locator (**b**) Max Locator.

Figure 4 (a) and (b) show the architectures for the min and the max locators. The min and the max locators consist of one comparator module and one multiplexer module. The function of the comparator is to check whether input 1 is less than input 2. The modules are reused again, thereby reducing area utilization. The inputs are 8 bits, and the selection line to the mux is 1 bit. The output from the comparator will act as the control line for the mux, from which the min and the max values are obtained.

**Fig. 5:**
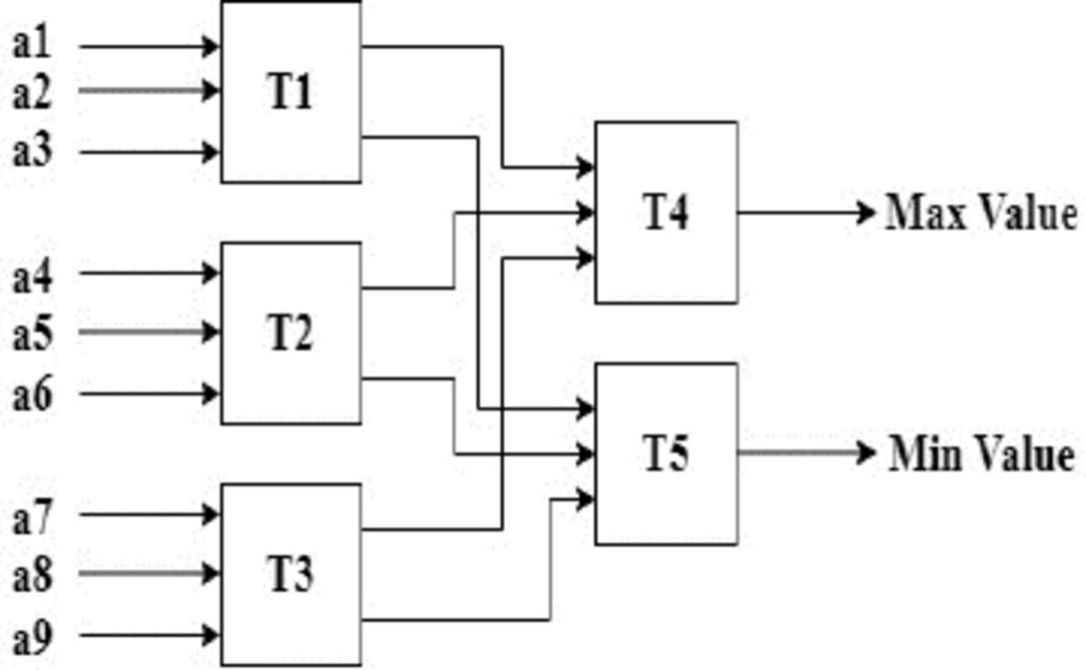
Tritonic sorter for a 3 × 3 window.

**Fig. 6:**
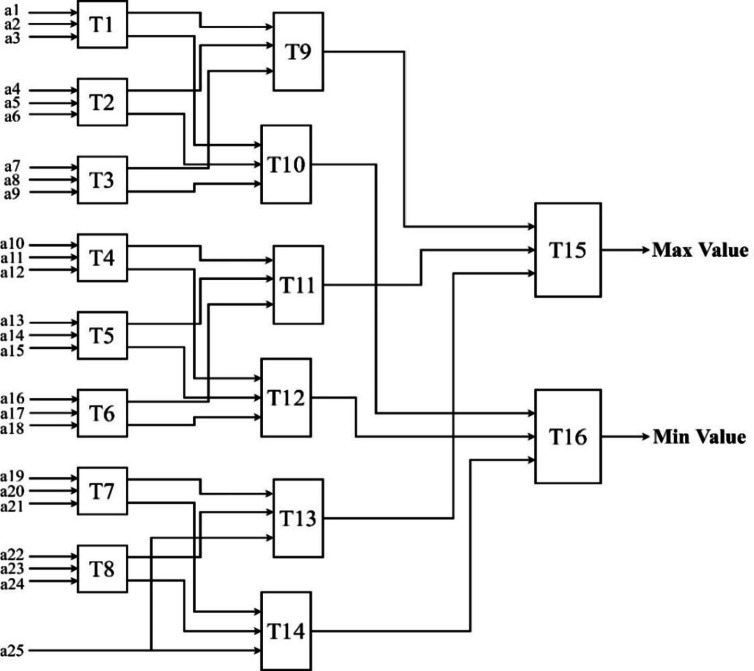
Tritonic sorter for a 5 × 5 window.

The Tritonic Sorter architectures for 3 × 3 and a 5 × 5 sliding are shown in Figs. 5 and 6, respectively.

A 5 × 5 sliding window will have 25 input values. The selection is because it provides a more accurate representation of the min-max values because it considers a larger neighborhood. The variations are smoothed, and a more stable estimate of the minimum and maximum values is provided. This architecture uses only 16 comparators to obtain the max and the min values, which is much less than other existing techniques. The maximum value is obtained from T15, and the minimum value is obtained from T16.

## Experimentation analysis

The image is read in the Vivado software tool via a text file. This text file contains the pixel values in hexadecimal format. The proposed Tritonic Sorter is compared with existing sorters^30–32^, and^33^, and the results are shown in Table 1. The proposed sorter gives better results regarding the utilization of the lookup table (LUT), Flip Flops, power, and delay.

**Table 1:** Comparison of various sorters for finding the minimum and maximum values in a window.

	Parallel Sorter	Odd-Even Sorter	Parallel Merge Sorter	Bitonic Sorter	Tritonic Sorter
LUT	425	**228**	331	300	357
Flip Flops	475	323	361	425	**100**
Power (W)	0.131	0.120	0.117	0.131	**0.115**
Delay (ns)	5.595	6.938	6.437	6.643	**5.234**

Table 2 shows the resource utilization of the MMM filter implemented using the existing sorters along with the proposed Tritonic Sorter. The table shows the proposed technique for area- and power-efficient use. Pipelining and parallel processing were incorporated to implement the proposed method and obtain improved results. The technique was implemented in the Vivado 2018.2 version of the ZedBoard Zynq Evaluation and Development Kit (xc7z020clg484-1). The kit is a robust platform for developing embedded systems that combine hardware and software components. The ZedBoard Zynq evaluation and development kit has an available I/O pin count of 484, IOBs of 200, an LUT of 53,200 and a Flip Flop of 106,400.

**Table 2:** Resource utilization of the MMM filter using various sorters.

	MMM Filter using Parallel Sorter	MMM Filter using Odd-Even Sorter	MMM Filter using Bitonic Sorter	MMM Filter using Tritonic Sorter
LUT Utilization	29,504	26,240	28,544	**25,088**
Flip Flops	**19,456**	29,280	32,544	19,968
Power (W)	0.196	0.218	0.228	**0.191**
Delay (ns)	**4.674**	7.037	6.991	5.496

### Performance metrics

For validation purposes, the performance metrics PSNR, structural similarity index measure (SSIM), mean square error (MSE), MAE, root mean square error (RMSE), Laplacian mean square error (LMSE) and universal quality index (UQI) are considered. The PSNR, SSIM and MSE are commonly used metrics to obtain information about the quality of an image. The LMSE metric is used to focus on edge preservation.

LMSE highlights areas of abrupt changes in intensity, which usually align with an image’s edges. By comparing the Laplacian-transformed images, the LMSE evaluates the degree of edge and fine detail preservation following filtering in a direct manner. It is a useful statistic for assessing an image’s performance because it is more susceptible to distortions and blurring. It is given by Eq. (5).

5 $$\:LMSE=\frac{\sum\:_{x=1}^{M}\sum\:_{y=1}^{N}{\left[L\left(X\left(x,y\right)\right)-L\left(Y\left(x,y\right)\right)\right]}^{2}}{\sum\:_{x=1}^{M}\sum\:_{y=1}^{N}{\left[L\left(X\left(x,y\right)\right)\right]}^{2}}$$

where M and N are the dimensions of the images, $\:X\left(x,y\right)$ is the pixel value at the position $\:\left(x,y\right)\:$in the original image, $\:Y\left(x,y\right)$ is the pixel value at the position $\:\left(x,y\right)$ in the filtered image, and *L()* is the Laplacian operator given by $\:L\left(X\left(x,y\right)\right)=X\left(x+1,y\right)+X\left(x-1,y\right)+X\left(x,y+1\right)+X\left(x,y-1\right)-4X\left(x,y\right)$.

The UQI considers brightness, contrast, and structural similarity when evaluating picture quality, offering a thorough and perceptually meaningful assessment. It is a reliable statistic for assessing overall image quality since it considers features of human eyesight that are significant for perceiving image quality. The equation for the UQI is given by Eq. (6):

6 $$\:UQI=\frac{4{\sigma\:}_{xy}{\mu\:}_{x}{\mu\:}_{y}}{({\mu\:}_{x}^{2}+{\mu\:}_{y}^{2})({\sigma\:}_{x}^{2}+{\sigma\:}_{y}^{2})}$$

where the means of the images X and Y are given by $\:{\mu\:}_{x\:}$and $\:{\mu\:}_{y}$, respectively; the variances of the images X and Y are $\:{\sigma\:}_{x}^{2}$ and $\:{\sigma\:}_{y}^{2}$, respectively, and the covariance of images X and Y is $\:{\sigma\:}_{xy}$.

### Results and discussion

Table 3 shows that the choice of a 3 × 3 window size for performing the median filtering preserves the fine details and edges and minimizes potential oversmoothing when using larger window sizes such as 5 × 5 or 7 × 7. The table shows the results from applying median filtering on the Baboon image with a noise density of 0.05. This shows that the 3 × 3 window performs better than the other windows.

**Table 3:** Median filtering for various window sizes.

Metric	3 × 3	5 × 5	7 × 7	9 × 9
PSNR	**23.4695**	21.2511	20.5585	20.1967
SSIM	**0.7057**	0.4807	0.3870	0.3416
MSE	**45.2350**	59.5235	64.4097	67.8614
RMSE	**17.1027**	22.0792	23.912	24.9291
MAE	**5.4147**	7.6906	8.5406	9.0723
LMSE	**78.6834**	88.7665	91.609	92.9222
UQI	**0.9120**	0.8482	0.8188	0.8008

Table 4 shows the MMM filter’s performance for various window sizes. The table shows the results of applying the MMM filter to the Baboon image with a noise density of 0.05.

**Table 4:** The MMM filtering for various window sizes.

Metrics	3 × 3	5 × 5	7 × 7	9 × 9
PSNR	24.0936	25.7294	**27.0120**	19.5917
SSIM	0.7498	0.8374	**0.8881**	0.5699
MSE	36.5092	23.0715	**17.1082**	18.7568
RMSE	15.9169	13.1846	**11.3747**	26.7274
MAE	4.4272	2.8634	**2.1424**	4.0229
LMSE	68.8142	50.1728	38.9192	**37.3086**
UQI	0.9242	0.9487	**0.9624**	0.8219

**Fig. 7:**
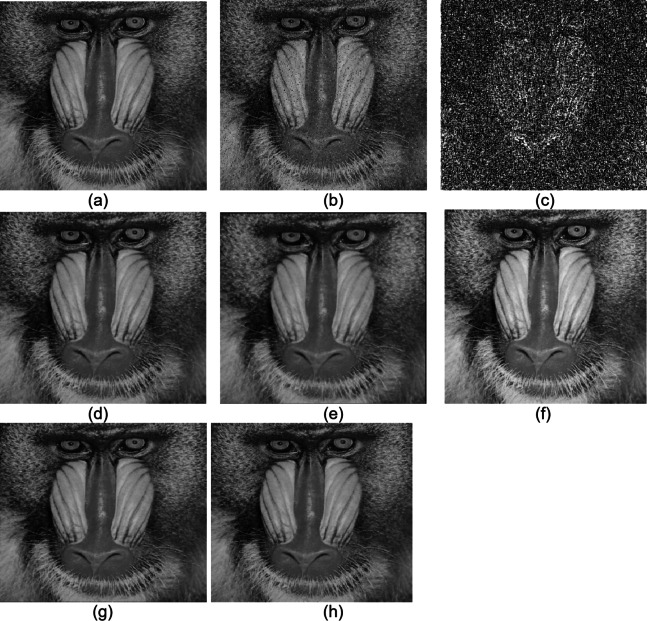
Baboon image **(a)** Original image **(b)** Noisy image **(c)** Min-Max sheet **(d)** MF **(e)** SMF **(f)** AMF **(g)** WMF **(h)** MMM filter.

**Fig. 8:**
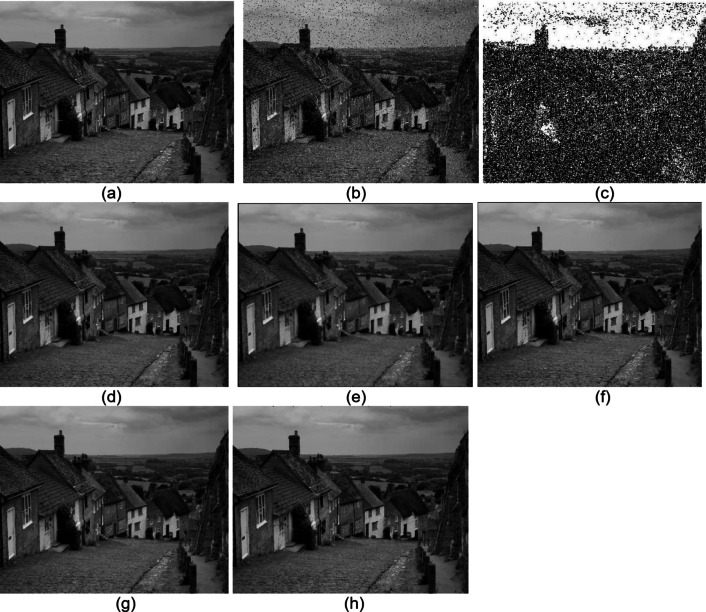
Goldhill image **(a)** Original image **(b)** Noisy image **(c)** Min-Max sheet **(d)** MF **(e)** SMF **(f)** AMF **(g)** WMF **(h)** MMM filter.

The proposed technique was implemented in MATLAB, and the results were compared with those of existing methods. Figures 7 and 8 show the Baboon and Goldhill images implemented using the median filter (MF), switching median filter (SMF), adaptive median filter (AMF), weighted median filter (WMF) and MMM filter. Figure 7’s original image is taken from^34^, and Fig. 8 is from^35^.

**Fig. 9:**
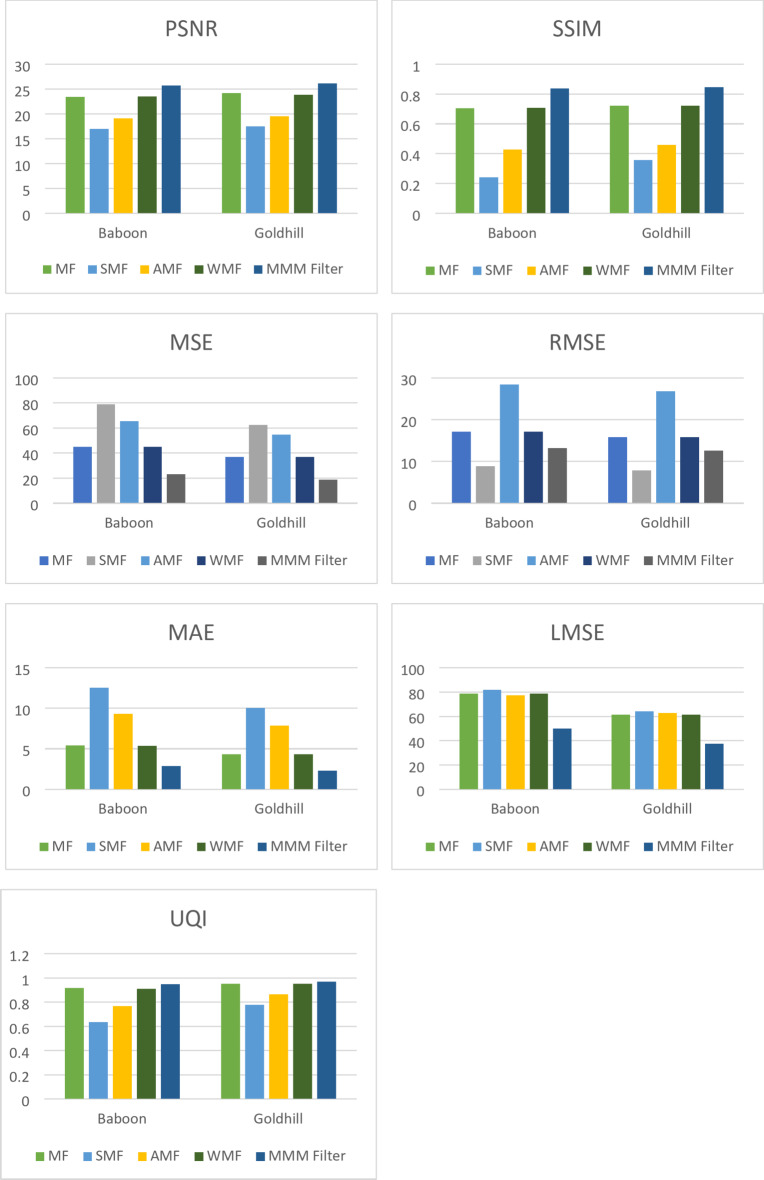
Performance metric comparison of the proposed technique with existing techniques.

Figure 9 shows the performance metric comparison results for the various filtering techniques on the Baboon and Goldhill images with a noise density of 0.05.

Tables 5 and 6 show the performance metric results for various filtering methods with different noise densities.

**Table 5:** Filtering methods performance for various noise densities on the Baboon image.

Filtering Methods		PSNR	SSIM	MSE	RMSE	MAE	LMSE	UQI
MF	0.01	23.6991	0.7144	44.2569	16.6565	5.2527	78.785	0.9162
	0.05	23.4637	0.7058	45.1318	17.1142	5.4011	78.6586	0.9116
	0.1	23.1435	0.24189	46.5001	17.7568	5.6251	78.4823	0.9055
SMF	0.01	17.0134	0.24189	78.8706	8.8809	12.4889	82.4395	0.6381
	0.05	16.9979	0.24128	78.8695	8.8808	12.5261	82.0053	0.6364
	0.1	16.9605	0.23922	79.1559	8.897	12.6294	81.3945	0.6342
AMF	0.01	19.1549	0.4343	64.9901	28.1058	9.2173	77.5092	0.7707
	0.05	19.0729	0.4278	65.4642	28.3722	9.3197	77.3795	0.7671
	0.1	18.9837	0.4203	65.7609	28.6652	9.4067	77.1334	0.7628
WMF	0.01	23.6896	0.7141	44.3149	16.6748	5.2659	78.7664	0.9162
	0.05	23.4831	0.7062	45.0341	17.0761	5.3812	78.6232	0.9118
	0.1	23.1155	0.6929	46.5016	17.8141	5.6218	78.5245	0.9049
MMM filter	0.01	25.7097	0.8285	23.8250	13.2146	2.9515	52.2343	0.9479
	0.05	25.7014	0.8369	23.0526	13.2273	2.8824	50.0746	0.9484
	0.1	25.2741	0.8295	25.3004	13.8943	3.1620	51.7542	0.9426

**Table 6:** Filtering methods performance for various noise densities on the Goldhill image.

Filtering Methods		PSNR	SSIM	MSE	RMSE	MAE	LMSE	UQI
MF	0.01	24.3444	0.7267	36.2720	15.4639	4.2132	61.4411	0.9535
	0.05	24.1516	0.7204	36.9479	15.811	4.3166	61.358	0.9516
	0.1	23.8228	0.7090	38.0157	16.421	4.5054	61.2263	0.9479
SMF	0.01	17.5208	0.35864	62.4996	7.9057	9.9913	64.3986	0.7810
	0.05	17.5047	0.35794	62.4981	7.9056	10.0209	64.1277	0.7801
	0.1	17.4592	0.35623	62.4528	7.9027	10.0582	63.6769	0.7789
AMF	0.01	19.6458	0.4651	54.3940	26.5613	7.7929	62.8125	0.8660
	0.05	19.5657	0.4596	54.8471	26.8075	7.8861	62.6742	0.8638
	0.1	19.5657	0.4543	55.1710	27.0696	7.9646	62.483	0.8616
WMF	0.01	24.3368	0.7265	36.2474	15.4774	4.2113	61.4444	0.9535
	0.05	23.8213	0.7206	37.0286	15.8194	4.3272	61.3458	0.9515
	0.1	23.8213	0.7091	37.8642	16.4239	4.4796	61.2164	0.9475
MMM filter	0.01	26.1223	0.8380	19.4313	12.6016	2.3593	38.6274	0.9694
	0.05	26.1584	0.8462	18.8759	12.5493	2.3133	37.5326	0.9701
	0.1	25.7632	0.8384	20.8445	13.1335	2.5678	39.8187	0.9665

## Conclusion

This paper addresses the growing complexity and interconnected nature of hardware and software design, proposing a more holistic approach to optimize performance and resource utilization. The paper proposes a modified Min-Max Median filter, incorporating a more advanced sorting mechanism to improve efficiency. The design incorporates two different window sizes: a k × k window to find the minimum and maximum values in each window and a 3 × 3 window to perform median filtering. To evaluate the effectiveness of the modified filter, the following performance metrics, PSNR, SSIM, MSE, RMSE, MAE, LMSE and UQI, were considered. The results of the proposed method indicated significant improvements in image quality, noise reduction, and edge preservation compared to existing techniques, making it an efficient tool for image processing. The paper introduces a Tritonic Sorter for efficiently finding the minimum, maximum, and median values. This sorter reduces the number of comparators required for sorting, optimizing both area and power. The Tritonic sorter resulted in a 27% reduction in area utilization compared to the traditional Bitonic sorters, making it highly beneficial for hardware-constrained environments. A 16.23% improvement in power consumption was achieved, which is critical in embedded systems. The proposed method is implemented using the ZedBoard Zynq evaluation and development kit and tested using Vivado 2018.2. This paper highlights the importance of integrating hardware-software co-design techniques to tackle the challenges of modern and complex systems.

## Data Availability

All data generated or analysed during this study are included in this published article.
